# Supplementary material for: A high-resolution mRNA expression time course of embryonic development in zebrafish
Source: eLife. 2017 Nov 16;6:e30860. doi: 10.7554/eLife.30860 (PMC5690287; doi:10.7554/eLife.30860)
Supplement: Supplementary file 6. [file elife-30860-supp6.zip › biolayout-clusters-files/Cluster208-genes.html]

Cluster208


# Cluster208: Genes

| Ensembl ID | Gene Name | Chr | Start | End | Biotype |
| --- | --- | --- | --- | --- | --- |
| ENSDARG00000016363 | her8a | 7 | 26158748 | 26165287 | protein\_coding |
| ENSDARG00000026053 | hltf | 2 | 25288406 | 25306368 | protein\_coding |
| ENSDARG00000030367 | metrn | 24 | 38053721 | 38068096 | protein\_coding |
| ENSDARG00000003940 | mtfr1l | 17 | 24699800 | 24708891 | protein\_coding |
| ENSDARG00000003607 | nr2f6a | 2 | 24718654 | 24730731 | protein\_coding |
| ENSDARG00000098679 | nup85 | 3 | 6557884 | 6591554 | protein\_coding |
| ENSDARG00000089637 | rnaseh2c | 14 | 35069009 | 35074278 | protein\_coding |
